# Dr. Christian A. Weinbach

**Published:** 1916-12

**Authors:** 


**Dr. Christian A. Weinbach,** Medical College of Mobile, Alabama 1894, Buffalo 1895, died at his home in Buffalo, Nov. 9, aged 42.

